# Variability in soil properties influencing pigeonpea ( *Cajanus cajana L.*) yield: a multivariate statistical analysis

**DOI:** 10.12688/f1000research.53095.3

**Published:** 2023-08-17

**Authors:** Rajesh N L, Narayana Rao K, Sathishkumar U, Wali V B, Basavaraj K, Rudramurthy H V, Desai B K

**Affiliations:** 1 Soil Science and Agricultural Chemistry, University of Agricultural Sciences Raichur, Raichur, Karnataka, 584104, India; 2 Soil Water Conservation Engineering, University of Agricultural Sciences Raichur, Raichur, Karnataka, 584104, India; 3 Agricultural Statistics, University of Agricultural Sciences Raichur, Raichur, Karnataka, 584104, India; 4 Agronomy, University of Agrcilutral Sciences Raichur, Raichur, Karnataka, 584104, India

**Keywords:** Soil-phase unit, Soil-plant relationship, Principal component regression analysis

## Abstract

**Aims: **The aim of the study was to reveal the variability in soil properties influencing pigeonpea ( *Cajanus cajana L.*) seed yield under semi-arid rainfed condition.

**Methods: **Soils were initially classified into series level and further these series were divided into soil-phase units. For two site years *viz.*, 2018-19 and 2019-20, surface soil samples from each soil-phase unit were collected before sowing of pigeonpea and subsequently crop growth parameters at critical stages were recorded.

**Results: **The principal component analysis with varimax rotation resulted in seven components for both the site years, having eigenvalues greater than one, explained more than 80% of the variability. The step wise linear regression analysis showed that the pigeonpea seed yield was linearly correlated with PC3 ( *p*<0.01), PC4 ( *p*<0.01) and PC7 ( *p*<0.05) of soil properties with R ^2^ = 0.679, during 2018-19. Whereas, during 2019-20, the seed yield was linearly correlated with PC1 ( *p*<0.01), PC3 ( *p*<0.01) and PC6 ( *p*<0.05) with R ^2^ = 0.677. In site year 1, the available P _2_O _5_, Fe, Zn, S, Cu, number of pods, surface soil moisture determined the yield. In site year 2, the available K _2_O, P _2_O _5_, Fe, Zn, S, clay, CEC and available water content determined the yield. All these variables together explain variability in yield.

## Introduction

Despite environmental perturbations, agricultural landscapes provide diverse ecosystem services and maintain crop yields ( John, 2020). The resilience of agricultural landscapes is underpinned by complex interactions between environmental factors (precipitations, temperature, pollutants, and sunlight) and agricultural inputs provided during crop cultivation ( FAO, 2013). Inherent soil properties, namely soil texture, type of clay, soil depth to bed rock and drainage class which result primarily from the soil-forming factors such as climate, topography, parent material, biota and time, influence the land suitability to cultivate a given crop to produce maximum yield ( Moore, 2001). Therefore, the relationship between soil and crop yield mostly depends on the prevailing climate and the landscape. The process of deriving spatial or statistical models to establish a relationship between soil and crop yield involves a systematic inventory of land resources, specifically soil (physico-chemical properties) and plant variables (growth and yield parameters). Efforts have been made using multivariate statistical analysis to reclassify the soil variability into spatial management units, based on principles of similarity which are analogous to crop yield.

Although a soil-phase is a unit of soil outside the system of soil taxonomy, understanding the variability of landscape and soil properties and their effect on crop yield is a critical component of site-specific and sustainable management interventions ( Juhos *et al.*, 2015). Therefore, soil-phase taxa at soil series level is a functional management unit delineated based on the differentiating soil (texture, pH, EC) and land surface (gravel, slope, erosion) characteristics. Pigeonpea is a multi-purpose leguminous crop that plays an important role in food security, maintenance of soil fertility through litter fall, nitrogen fixation, provision of fodder for livestock and fuel for small-scale farmers in subsistence-agriculture ( Egbe & Kalu, 2006). It improves soil organic matter content by leaf fall at the time of maturity and its deep tap root system breaks the plough pans and improves the soil structure. Hence, pigeon pea is often called as “Biological plough”. Extensive ground cover by pigeonpea prevents soil erosion due to wind and water which encourages the infiltration of rainwater and smothers the weeds.

Multivariate analysis would be most appropriate to understand the specific variables influencing crop yield. Principal component analysis (PCA) is an extremely powerful analytical technique and can often indicate which variables in a data set have a significant effect on the response variables, and which are not significant ( Hair *et al.*, 2003). PCA can simplify the structure of a set of variables by replacing those with a few uncorrelated linear combinations of original variables. As soil parameters are correlated with each other due to their multicollinearity, it makes sense to examine them jointly ( Zsigrai, 1999); therefore, there is the need to evaluate the correlation among the soil properties and crop growth parameters using PCAPCA is a mathematical linear transformation of the original variables, which accounts for the maximum share of the variability present in the original set of variables, with a minimum number of composite variables and makes no assumption of a model ( Jolliffe & Cadima, 2016). Therefore, stepwise linear regression analysis to check the significance of soil properties and plant growth attributes is important for better understanding of the maximum share of variability present in the original set of variables. Further, these derived significant variables could be considered as primary targets for interventions to address the site-specific soil variability and improve the crop yield.

## Methods

### Study setting

**Figure 1.**
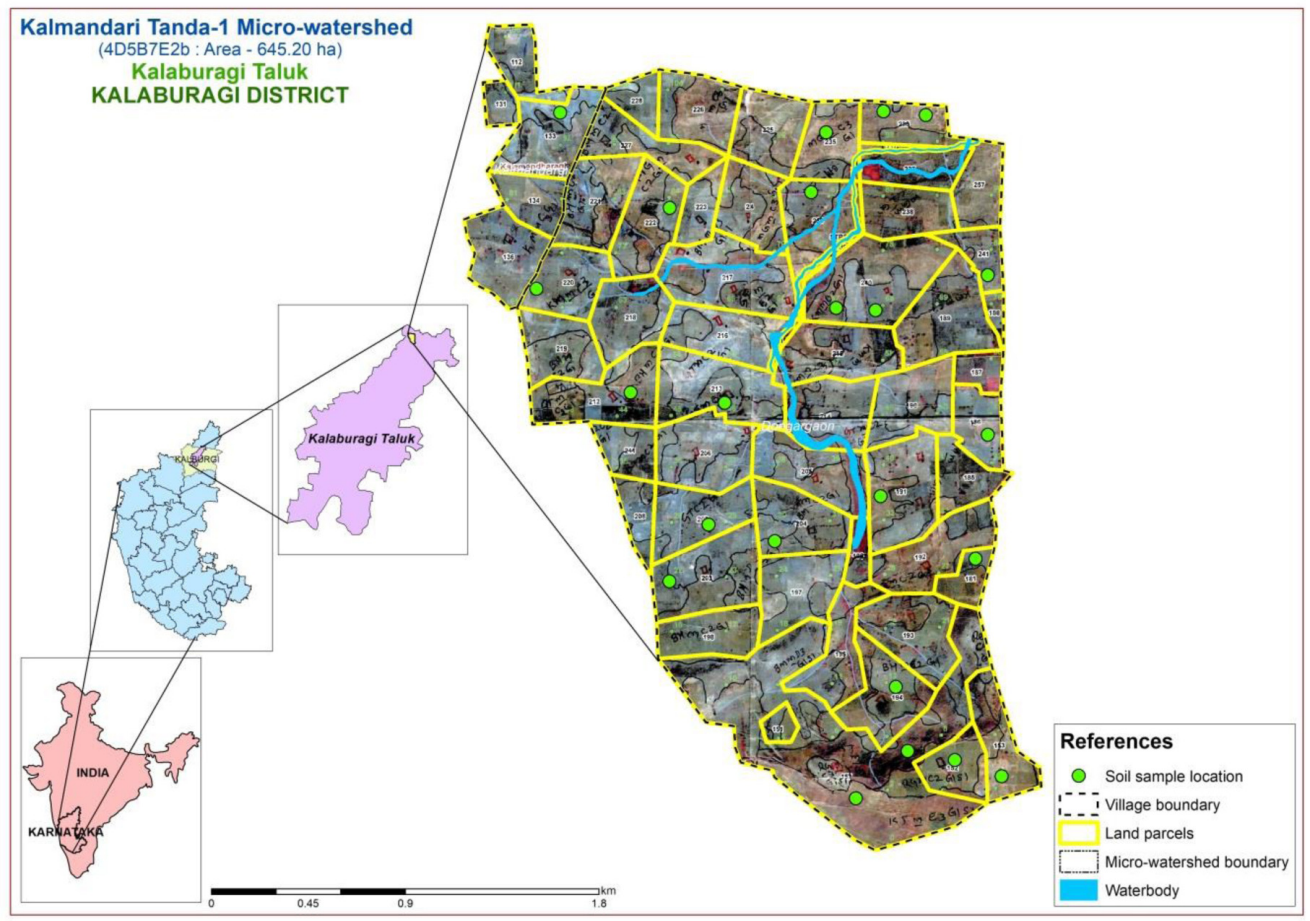
Location map with soil physiography interpreted imagery of LISS IV merged Cartosat-1.

The study area was located in the Northern dry zone of Karnataka State, India ( Figure 1), between latitudes and longitudes of 17°40’17.956’’ N - 76°59’46.836” E and 17°38’12.689” N - 77°1’17.049” E, covering 645.20 ha, at an altitude of 511 - 637.86 m above mean sea level. The study was planned for two site years *viz*., during kharif (cropping season between June to October) 2018–2019 and kharif 2019–2020, to assess the variability in surface soil properties for each soil phase and crop growth parameters influencing pigeonpea yield.

**Figure 2.**
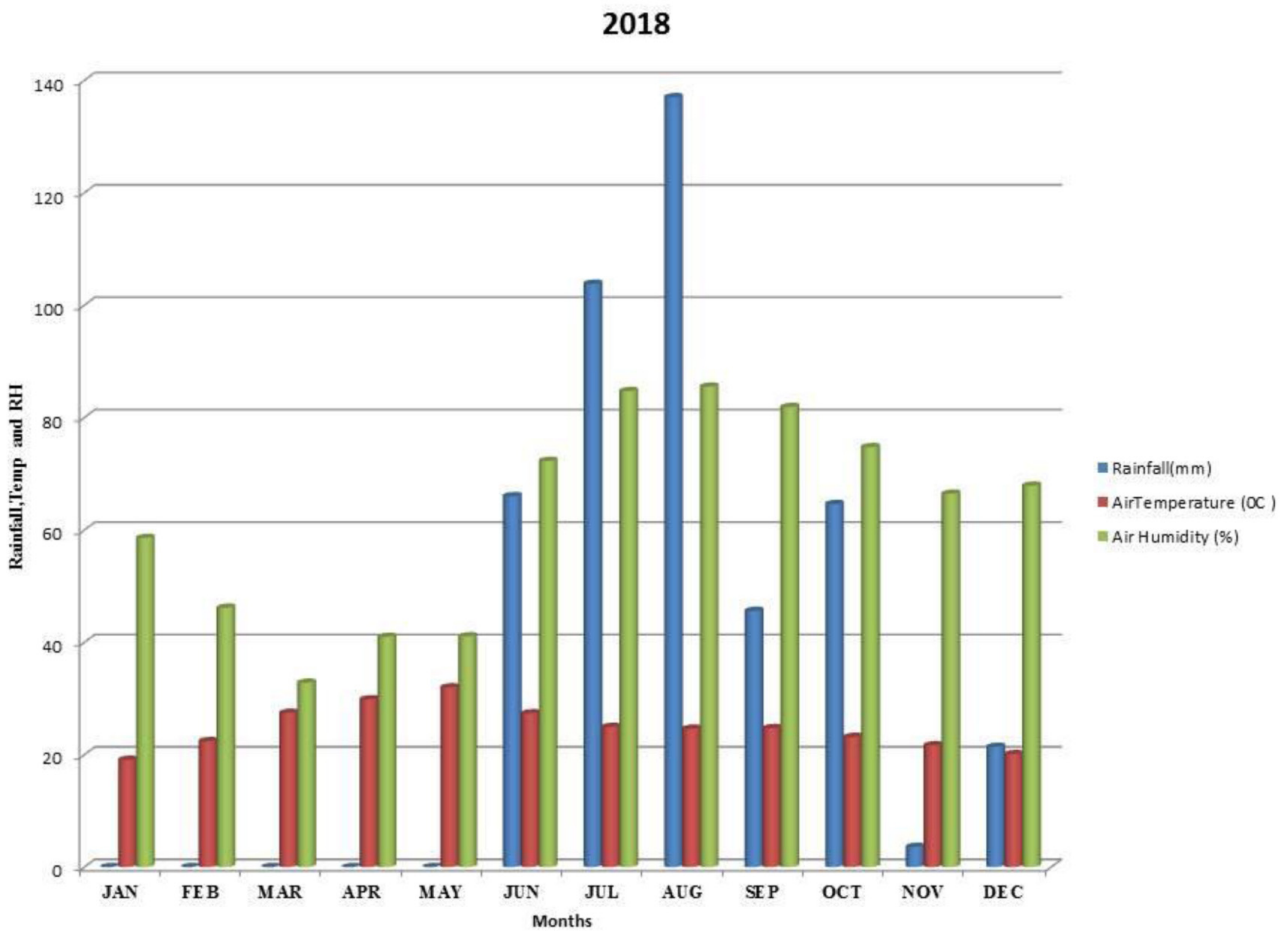
Mean monthly weather parameters of Kalmandari Tanda-1 MWS during 2018.

**Figure 3.**
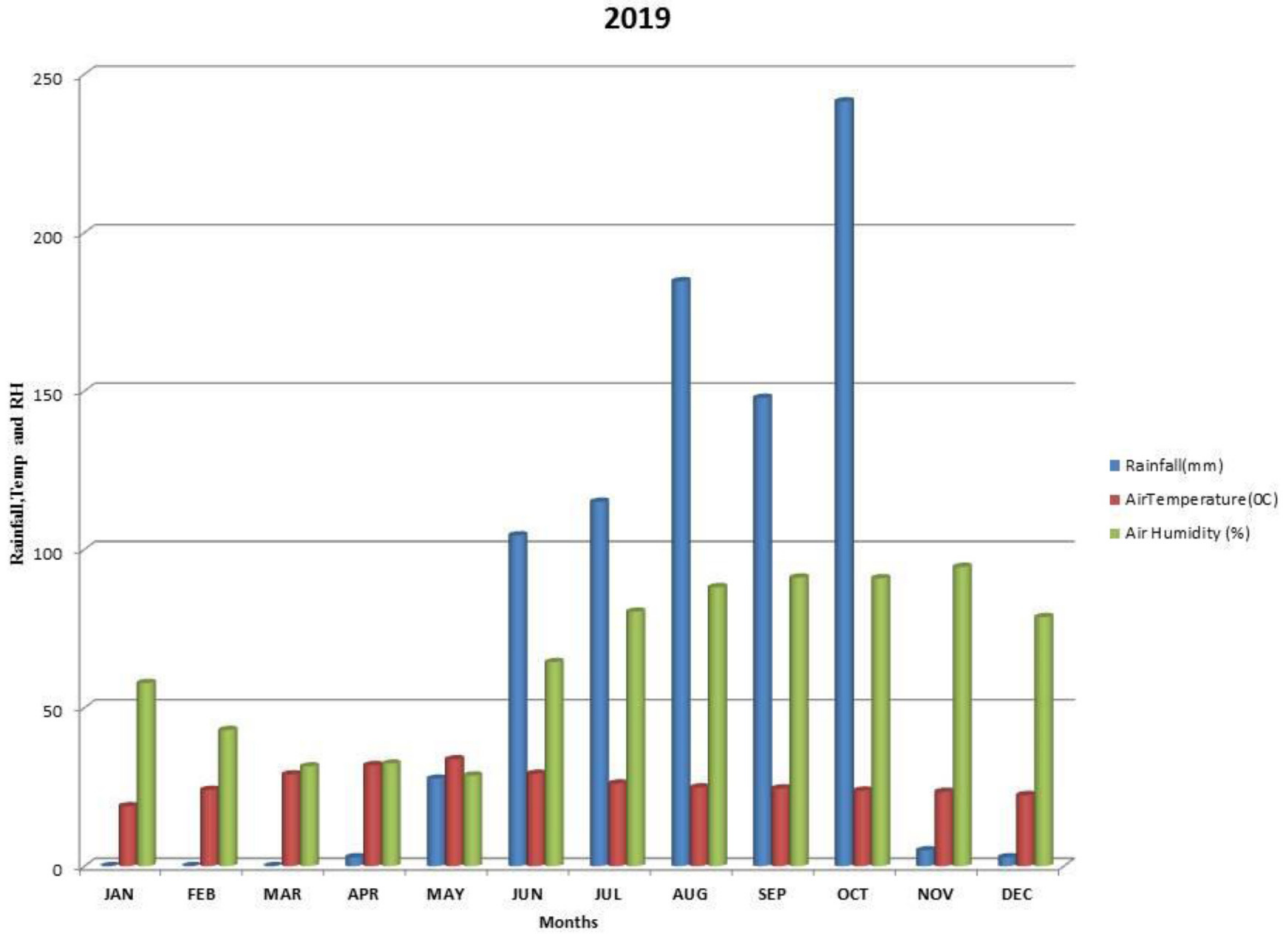
Mean monthly weather parameters of Kalmandari Tanda-1 MWS during 2019.

Kalmandari Tanda-1 micro-watershed (MWS) reported mean monthly minimum and mean monthly maximum temperatures of 19.08°C and 31.97°C, respectively, during January 2018 and May 2018, whereas the reported values for these variables were respectively 18.89°C and 33.7°C for January 2019 and May 2019. The average annual rainfall measured by Kalmandari Tanda-1 MWS was 442 mm and 831.50 mm in 2018 and 2019, respectively ( Figure 2 and Figure 3).

### Experimental procedures

**Figure 4.**
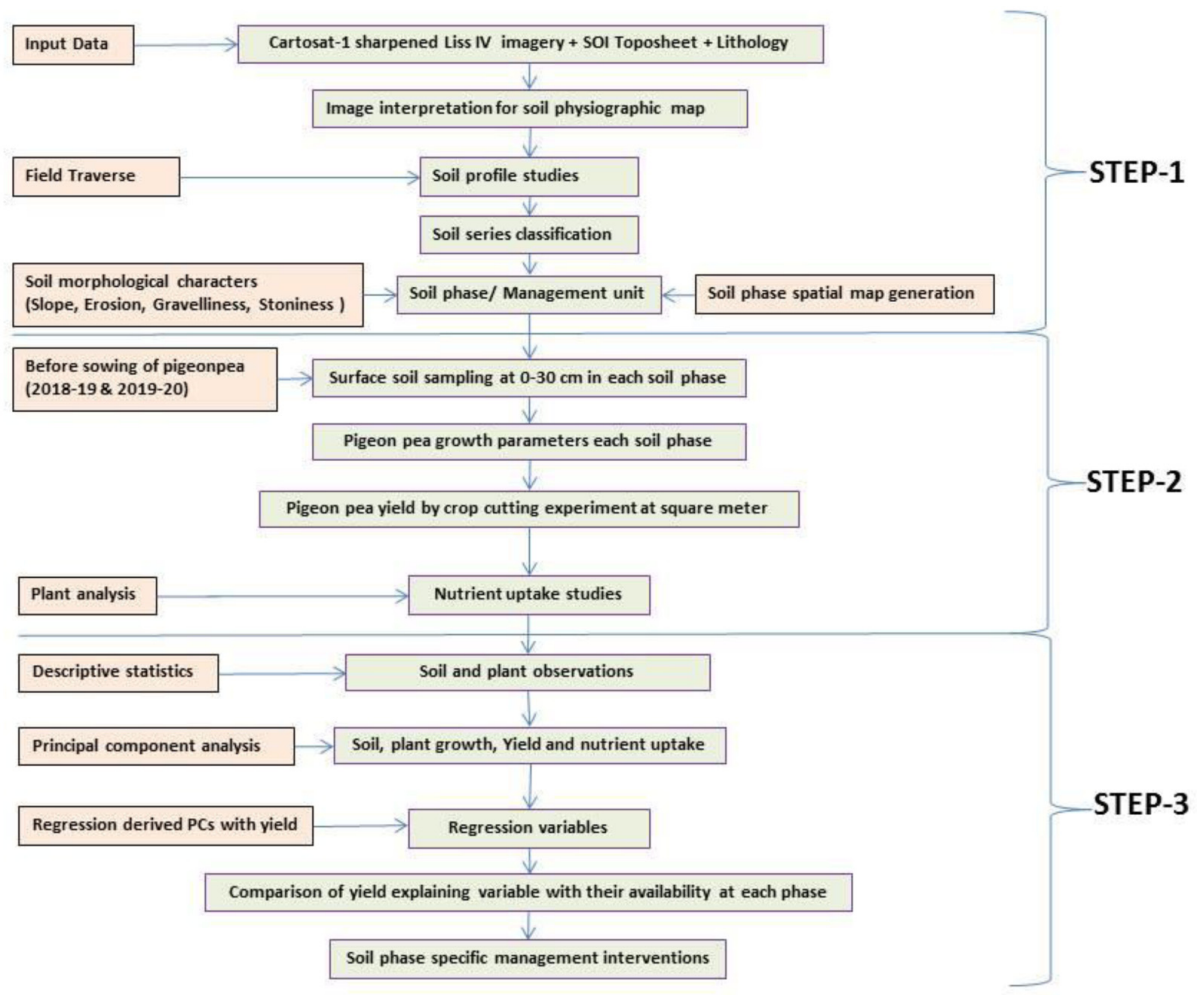
Methodological workflow.

A comprehensive methodological workflow adopted in this study is presented in Figure 4. To divide the entire micro-watershed into different soil phase units, a detailed land resource inventory was initially carried out at a 1:8000 scale, using Indian Remote Sensing Satellite-P6 (IRS P6) Cartosat-1 merged LISS-IV satellite imagery (2.5 m spatial resolution) as base map to interpret the soil physiographic unit map ( Figure 1). The soil (soil profile depth, number of horizons, soil color, soil structure, texture, consistency, presence of carbonates, and soil pH) and site (slope, erosion, drainage, runoff, gravelliness, stoniness, presence of rocks on surface, lithology/parent material and current land use) characteristics were recorded for all soil profile sites on a standard *pro forma* (Pedon description form which consists of a list of soil and site parameters that need to be recorded during a field study. A copy is provided in the Data availability section (see *Extended data,* Rajesh, 2021)) as per the guidelines given in *Field Guide for Land Resources Inventory* ( Natarajan *et al*., 2016).

**Figure 5.**
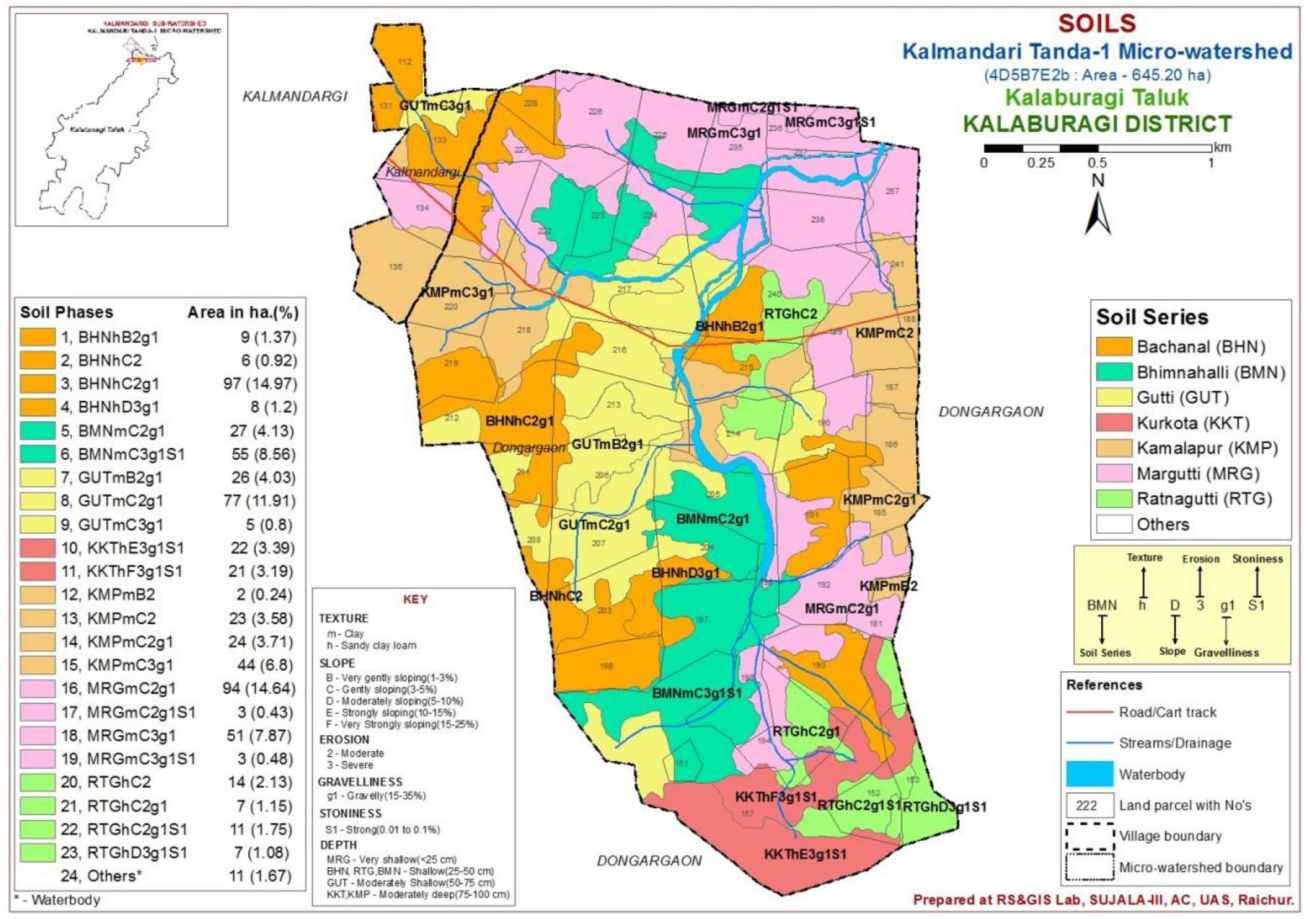
Soil phases of Kalmandari Tanda -1 micro watershed.

In this micro-watershed, both Vertisol and Alfisol soils were found, which are soils derived from basalt and basalt-laterite intrusion parent materials, respectively ( Government of India, 2008). Pedons that showed similar soil profile horizons in soil color, soil texture, soil pH, and consistence, mineral and chemical compositions were grouped into soil series ( FAO, 2006) and further, these soil series were divided into 23 soil phases ( Figure 5), based on surface characteristics with respect to soil texture, slope, erosion and gravelliness.

A total of 462 surface soil samples (0–30 cm) were collected in two site years. Each year, before sowing of pigeon pea, 231 soil samples were collected from the same locations (recorded using Trimble Juno 3D, a hand-held GPS) of each soil phase of the micro-watershed. Physico-chemical properties of all collected surface soil samples were analyzed using standard procedures. Erosion hazards were judged through the visible soil erosion method, by assessing the presence of rills and gullies within a field, as well as their associated deposits ( Evans, 2013). Soil texture was evaluated using the feel method, and slope with the help of a dumpy level. Organic carbon (OC) was determined using the Walkley & Black (1934) wet oxidation method. Available N was determined by a modified alkaline potassium permanganate method as described by Subbiah & Asija (1956). Available P _2_O _5_ was determined using Olsen’s method. Available K _2_O was estimated using a flame photometer after extraction with ammonium acetate. Soil reaction (pH) was determined in 1:2.5 soil water suspensions using a glass electrode ( Piper, 1966). Electrical conductivity was measured in the soil water (1:2.5) suspension using a conductivity bridge ( Jackson, 1973). The level of free calcium carbonate ions in soil samples was determined using a rapid titration method with standard HCl ( Piper, 1966).

Cationic micronutrients like iron, copper, manganese and zinc were extracted using diethylene triamine penta acetic acid (DTPA, 0.005 M and 0.01 M CaCl _2_ + 0.1N tri-ethanol-amine at pH 7.3), and the concentration was measured using an atomic absorption spectrophotometer (ContrAA 700 Make) as outlined by Lindsay & Norvell (1978). Available boron in soil was estimated with a colorimetric method using hot water as extractant and expressed in mg kg ^-1^ ( Page *et al.*, 1982). Available sulphur in soil was extracted with CaCl _2_.2H _2_O (0.15%), and the extract was reacted with barium chloride crystals. The intensity of the resulting turbidity was measured using a spectrophotometer at a wavelength of 420 nm ( Jackson, 1973)

The bulk density was estimated using Keen’s cup method for the disturbed soils ( Piper, 1966). Particle size distribution of soil samples was determined using the "International Pipette" method ( Piper, 1966); the soil’s cation exchange capacity (CEC) was determined by equilibrating the soil with 1N sodium acetate solution using a flame photometer, to calculate the CEC. The exchangeable sodium percentage (ESP) was calculated by dividing the exchangeable sodium by CEC and exchangeable bases (Ca, Mg, and K, Na), which were measured by titration and flame photometer respectively.

Pigeon pea growth and yield parameters were recorded using a crop cutting experiment, where observations were regularly made in the farmer’s field at specific locations of the study area and at different crop growth stages. The growth parameters *viz.*, plant height (in centimeter), number of branches per plant, and leaf area index (AccuPAR Ceptometer model LP-80) were recorded for each soil phase. Five pigeon pea plants were labeled in a 1 m ^2^ area of each soil phase unit and the growth parameters were recorded at 30, 60, and 90 days after sowing (DAS), as well as during the crop harvesting stage. Similarly, pigeon pea yield parameters, namely the number of pods per plant and grain yield (kg ha ^-1^), were recorded at the harvest stage and after the harvest respectively. Details of pigeon pea varieties and fertilizer doses (urea and di-ammonium phosphate manufactured by Zuari Agro Chemical Ltd., India) used are given in the *Extended data* ( Rajesh, 2021). Meanwhile, soil moisture and available water content (AWC) at each soil-phase were measured using a theta probe (Stevens Water Monitoring Systems Inc, S/N 238825) and pressure plate membrane apparatus (SOILMOISTURE Equipment Corp, Model #1500F2), respectively.

### Statistical analysis

Descriptive statistics and principal component regression analysis of soil and plant parameters were carried out in SPSS v.16. An assessment of the normality of the data is pre-requisite for PCA. A Z-scale test was applied for normality test using skewness and kurtosis (for n < 300). The distribution is approximately normal if skewness and kurtosis are between -1 and 1. The right and left skewed data ( Table 1 and Table 2) were subjected to logarithmic and square root transformation to improve the normality. These transformed variables were subjected to a PCA with varimax rotation ( Ayoubi *et al.*, 2009; Cox *et al*., 2003; Shukla *et al*., 2004b). The descriptive statistics of physico-chemical properties of soil and plant growth parameters are presented in Table 1 and Table 2, respectively.

**Table 1.** Descriptive statistics of physico-chemical properties of surface soil (before sowing) during 2018–2019 and 2019–2020.

Parameters	2018–19						2019–20					
	Min	Max	Mean	SD	Skewness	Kurtosis	Min	Max	Mean	SD	Skewness	Kurtosis
pH	6.08	8.60	6.75	0.57	1.849	4.416	5.88	7.78	6.63	0.44	0.711	0.650
EC (dSm ^-1^)	0.13	0.50	0.31	0.10	-0.101	-0.862	0.11	0.49	0.27	0.09	0.643	0.230
CaCO _3_ (%)	4.23	7.80	5.55	0.77	1.067	2.272	5.23	9.00	6.67	0.84	0.840	1.520
OC (%)	0.32	0.74	0.55	0.09	-0.519	1.120	0.27	0.69	0.50	0.09	-0.520	1.120
Available N (kgha ^-1^)	144.00	333.00	248.05	40.68	-0.512	1.144	121.50	310.50	225.55	40.68	-0.510	1.140
Available P _2_O _5_ (kgha ^-1^)	27.50	51.80	40.60	7.28	-0.273	-0.899	20.18	44.68	30.65	6.31	0.882	0.270
Available K _2_O (kgha ^-1^)	311.98	459.39	371.25	45.74	0.447	-0.914	302.42	449.83	361.69	45.74	0.453	-0.910
Available S (kgha ^-1^)	5.00	11.60	8.68	2.083	-0.270	-1.098	7.00	13.60	10.69	2.08	-0.274	-1.100
Available Zn (ppm)	0.38	0.99	0.65	0.15	0.523	-0.181	0.22	0.75	0.44	0.18	0.692	-0.910
Available Fe (ppm)	1.30	4.80	3.21	1.09	-0.363	-1.400	1.15	4.65	2.97	1.12	-0.181	-1.570
Available Cu (ppm)	0.65	1.31	0.94	0.18	-0.06	-0.793	0.55	1.21	0.85	0.19	-0.060	-0.790
Available Mn (ppm)	7.59	14.70	11.057	2.174	0.051	-0.936	6.99	14.50	10.44	2.55	0.080	-1.440
Exch. Ca [Cmol(p+)kg ^-1^]	14.80	28.54	21.87	4.065	0.009	-1.174	16.80	30.54	23.88	4.07	0.010	-1.170
Exch. Mg [Cmol(p+)kg ^-1^]	4.23	8.15	6.26	1.14	-0.007	-1.105	5.23	9.15	7.25	1.15	0.030	-1.180
Available B (ppm)	0.30	0.50	0.43	0.05	-1.150	1.032	0.19	0.52	0.37	0.10	-0.350	-1.380
Exch. Na [Cmol(p+)kg ^-1^]	0.78	1.89	1.12	0.28	1.326	1.244	0.74	1.85	1.09	0.28	1.330	1.240
ESP (%)	2.61	5.55	3.50	0.87	1.335	0.871	2.43	5.28	3.28	0.84	1.340	0.890
CEC [Cmol(p+)kg ^-1^]	27.59	34.75	32.20	2.35	-0.482	-1.498	28.54	35.70	33.16	2.35	-0.480	-1.503
Sand (%)	18.62	55.20	38.87	9.46	-0.047	-0.498	18.62	55.20	38.87	9.46	-0.052	-0.500
Silt (%)	15.00	27.00	22.14	3.32	-0.869	-0.100	15.00	27.00	22.14	3.32	-0.871	-0.101
Clay (%)	24.80	55.13	37.96	8.48	0.068	-1.136	24.80	55.13	37.97	8.49	0.072	-1.140
BD (Mgm ^-3^)	1.25	1.36	1.30	0.03	0.285	-1.424	1.21	1.38	1.30	0.05	-0.260	-0.560
PWP (%)	11.45	16.12	13.22	1.25	0.746	-0.038	11.45	16.12	13.27	1.34	0.610	-0.630
FC (%)	33.45	49.74	41.15	4.99	0.069	-1.381	25.21	39.74	32.34	3.94	0.060	-0.760
AWC (%)	21.00	36.14	27.93	4.72	0.260	-1.117	13.76	26.94	19.07	3.92	0.570	-0.610

**Inference from multivariate analysis:** During 2018–19, yield contributing variables such as soil available P _2_O _5_, Fe, Zn, Cu and soil moisture (% AWC) and whereas during 2019–20 soil available K _2_O, S, Fe, Zn, Mn, Ca, Mg, CEC, clay, and available water content (AWC), a higher availability of these variables will be more beneficial. Exch: exchangeable; ESP: exchangeable sodium percentage; CEC: cation exchange capacity; BD: bulk density; PWP: permanent wilting point; FC: field capacity.

**Table 2.** Descriptive statistics of plant growth parameters at Kalmandari Tanda-1 MWS during 2018–19 and 2019–2020.

Parameters	2018–19						2019–20					
	Minimum	Maximum	Mean	Std. Deviation	Skewness	Kurtosis	Minimum	Maximum	Mean	Std. Deviation	Skewness	Kurtosis
Plht at 30 DAS	21.00	34.00	26.96	2.99	0.17	4.416	25.00	38.00	31.13	3.17	0.16	-0.23
Plht at 60 DAS	65.00	92.00	77.04	5.19	0.22	-0.862	70.00	97.00	82.04	5.21	0.23	3.41
Plhtat 90 DAS	100.00	118.00	108.22	6.52	0.40	2.272	103.00	121.00	111.13	6.28	0.38	-1.40
Plhtat harvest	110.00	134.00	122.52	6.93	-0.18	1.120	111.00	136.00	124.57	6.98	-0.26	-0.37
Brat 30 DAS	3.00	5.00	3.96	0.71	0.06	1.144	4.00	6.00	4.96	0.74	0.07	-0.85
Brat 60 DAS	7.00	10.00	8.78	0.90	-0.34	-0.899	9.00	12.00	10.78	0.912	-0.35	-0.46
Brat 90 DAS	9.00	13.00	10.96	1.15	0.49	-0.914	13.00	17.00	15.17	1.27	-0.06	-1.06
Brat harvest	11.00	15.00	13.17	1.27	-0.06	-1.098	14.00	18.00	16.17	1.33	-0.06	-1.06
LAIat 30 DAS	0.31	1.02	0.68	0.20	-0.27	-0.181	0.44	1.15	0.83	0.19	-0.40	-0.42
LAIat 60 DAS	0.93	2.04	1.74	0.25	-1.53	-1.400	1.14	2.25	1.95	0.25	-1.53	3.76
LAIat 90 DAS	2.04	3.91	2.80	0.55	0.80	-0.793	2.22	3.89	2.88	0.43	0.88	0.74
LAIat harvest	0.64	1.85	1.07	0.30	1.31	-0.936	0.74	1.95	1.17	0.365	1.35	1.60
Podat harvest	45.00	64.00	54.48	5.03	-0.08	-1.174	49.00	73.00	59.74	5.96	0.03	-0.17
crop yield	1207.00	1394.00	1300.90	51.01	-0.30	-1.105	1325.00	1496.00	1421.00	47.69	-0.47	-0.60

**Note**: Plht: Plant height, Br: Number of branches, LAI: Leaf area index, DAS: Days after sowing. **Inference from multivariate analysis:** In both the site years “number of pods at harvest” have contributed to the yield and is the one which explains yield variability among plant growth & yield parameters

### Multivariate analysis

PCA was used as a data reduction technique. The independent variables such as soil properties (soil pH, EC, CaCO _3_, OC, available N, P _2_O _5_, K _2_O, S, Zn, Fe, Cu, Mn, B, exchangeable Ca, Mg, Na, ESP, CEC [Cmol(p ^+^)kg ^-1^], Sand [%], Silt [%], Clay [%], bulk density [Mgm ^-3^], permanent wilting point [PWP in %], field capacity [FC in %], AWC [%]) and plant growth parameters (plant height, number of branches per plant, LAI, number of pods at harvest stage) were subjected to a PCA; variables with factor loadings greater than the measured Kaiser-Meyer-Olkin (KMO) values, were chosen as highly correlated variables from the derived principal components (PCs) with eigenvalues greater than one ( Suryanarayana & Mistry, 2016). The KMO test is a measure of sampling adequacy and factorability. The KMO tests whether the partial correlations within the data are close enough to zero to suggest that there is at least one latent underlying factor among variables ( Vijayamohanan Pillai & Asalatha, 2020). As a KMO value of < 0.5 is unacceptable ( Hair *et al.*, 2003), the results of the PCA with a KMO > 0.5 loadings indicate that the chosen correlation matrix was appropriate for factor analysis.

The Kaiser Meyer Olkin (KMO) test is given by;

Equation 1: $MO_{j}=\frac{\sum_{i\neq j}r_{ij}^{2}}{\sum_{i\neq j}r_{ij}^{2}+\sum_{i\neq j}U}$

Where, r = [r _ij_] - Correlation matrix

            U = [u _ij_] - Partial covariance matrix

The eigenvalue is the variance explained by a component or factor and is denoted by lambda.

Equation 2: $\lambda \kappa =\sum_{i=1}^{n}{Α}^{2}ik$

Where, A ^2^ *ik* is the factor loading for variable *i* on component *k,* and *n* is the number of variables. A low eigenvalue represents a lower contribution to the variance in the analyzed set of variables. The component matrix table shows the component loadings, which represent the correlations between the variables and the components. As a rule of thumb, the larger the size of the component loading for a variable, the more significant the variable is in the interpretation of the component ( Nargundkar, 2005). The first component is generally more highly correlated with the variables than the second components, and so on. A varimax rotation enhances the interpretability of the uncorrelated components; therefore, only the outcomes of the rotated component matrix are presented in our results.

### Linear principal component regression (LPCR) analysis

The derived PCs were subjected to a stepwise linear regression analysis, which is an alternative to multiple regressions analysis. In LPCR, instead of regressing the dependent variables on the explanatory variables directly, the principal components of the explanatory variables were used as regressors, each time removing the weakest correlated variable. The highly correlated and significant soil and plant independent/explanatory variables from each PC were fitted to the dependent variable (pigeon pea crop yield). Further linear equations were drawn separately.

## Results and discussion

**Figure 6.**
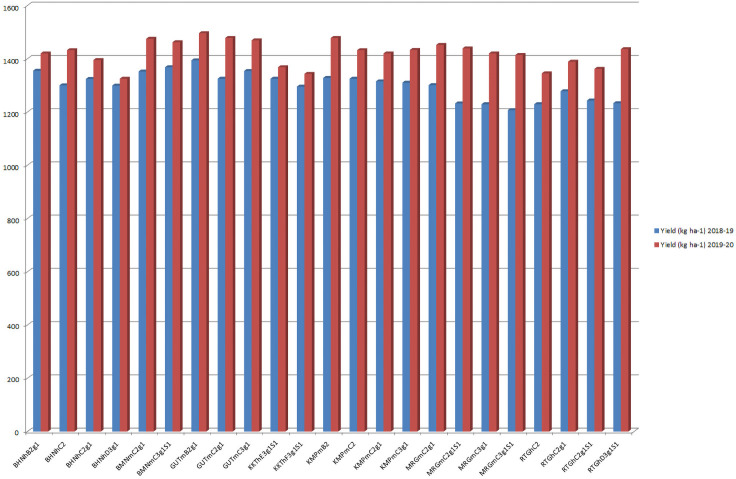
Pigeon pea crop yield (kg.ha ^-1^) during 2018–19 and 2019–20.

The soil phases GUTmB2g1, GUTmC2g1 and GUTmC3g1 from the Gutti soil series ( Figure 6) showed maximum crop yield (pooled) during *kharif* (June to October) 2018–19 (4073 kgha ^-1^) and *kharif* 2019–20 (4443 kgha ^-1^). The GUTmB2g1 soil phase prevailed on gentle slopes (1–3%) could give rise to a maximum yield of 1394 and 1496 kgha ^-1^ during *kharif* 2018–19 and 2019–20 respectively. However, of the two site years, crop yield was greater during *kharif* 2019–2020, due to a greater available soil moisture than that of *kharif* 2018–2019. The KKThE3g1S1 and KKThF3g1S1 soil phases of Kurukota series prevailed on stronger slopes (10–15 %), showed severe erosion (3), and led to a low yield in both *kharif* 2018–19 (respectively 1325 and 1295 kgha ^-1^) and *kharif* 2019–20 (respectively 1368 and 1343 kgha ^-1^). Thus, these soil phases would need intensive care and management, including fertilizer input and soil and water conservation strategies, so as to boost crop productivity.

### PCA & LPCR to assess the influence of soil properties on pigeon pea yield

Seven components were extracted with total cumulative variances of 82.30% and 80.75% during 2018 ( Table 3) and 2019 ( Table 4), respectively. The principal component 1 (PC1) in each site year was identified as soil available nutrient content with high loadings of K _2_O (0.843 and 0.764, respectively), Mn (0.870 and 0.882 respectively), Ca (0.845 and 0.900), and Mg (0.835 and 0.919 respectively). The presence of these adsorbed cations on negatively charged clay surfaces (supported by high loadings of clay content (0.905and 0.929) contributed to significantly high CEC loadings (0.897 and 0.937) ( Schoonheydt *et al*., 2018). High clay content supported high available water content with loadings of 0.822 and 0.625 during 2018-19 and 2019-20 respectively. Therefore, sand (-0.858 and -901) and bulk density (-0.927 and -0.743) are negatively correlated in PC1 of both the site years. Though the initial mean available water content is high (27.93 %) in soils during 2018–19 ( Table 1), the scarcer rains in subsequent months during 2018–19 ( Figure 2) has inferred that there was no sufficient soil moisture to dissolve the soil nutrients to make it readily available to pigeonpea plant when compared to the rains of 2019–20 ( Figure 3). In 2018, PC2 was identified as humification and vegetative growth with high loadings of OC and available N. The high loading of EC during 2018 may be due to the retention of dissolved salts associated with low precipitation. In 2019, because of higher precipitation levels ( Figure 3) the results of PC2 converged towards enhanced calcification with high loadings of CaCO _3_ (0.793) due to dissolution of CaCO _3_ in the presence of CO _2_ ( Neina, 2019) which is normally found at ten times greater levels in soil (0.3 % CO _2_) when compared to the CO _2_ concentration (0.03%) in the atmospheric air above the soil. Further, PC2 in site year 2 was also identified as qualitative vegetative growth with high loadings of Cu (0.764), which enhances plant photosynthesis; this was also supported by an increased number of branches (0.595) per plant at the time of harvest. PC3 in 2018 and 2019 was identified as improved enzyme activity and pod formation stage, which was supported by high loadings of Fe (0.919 and 0.889), Zn (0.846 and 0.735) and number of pods (0.736 and 0.605) at harvest.

**Table 3.** Factor loadings of soil properties (before sowing during June 2018).

Parameters	PC1	PC2	PC3	PC4	PC5	PC6	PC7
Eigen value	7.425	3.132	2.678	1.979	1.912	1.824	1.626
% of Variance	29.700	12.528	10.714	7.915	7.647	7.298	6.504
Cumulative %	29.700	42.228	52.942	60.857	68.504	75.802	82.306
**KMO=0.611**							
pH	0.346	-0.067	-0.101	-0.150	**0.752**	-0.118	0.031
EC	0.059	**0.858**	-0.045	-0.063	0.081	-0.205	-0.019
OC	0.379	**0.840**	0.100	0.054	-0.187	-0.110	0.146
CaCO _3_	-0.035	0.073	0.135	-0.105	-0.065	**-0.781**	0.122
N	0.381	**0.839**	0.100	0.056	-0.185	-0.107	0.148
**P _2_O _5_**	-0.110	0.053	-0.056	**0.801**	-0.119	-0.121	-0.021
K _2_O	**0.843**	0.040	-0.019	-0.146	-0.053	-0.036	-0.267
**S**	-0.050	0.010	-0.003	-0.442	0.254	0.158	**-0.688**
**Fe**	0.050	-0.080	**0.919**	-0.020	0.010	0.048	0.144
**Zn**	0.002	0.037	**0.846**	0.024	-0.039	-0.123	-0.159
Mn	**0.870**	0.078	0.031	0.059	0.085	-0.171	0.115
**Cu**	0.111	0.350	0.028	-0.401	0.029	0.141	0 **.682**
B	-0.173	-0.275	0.081	-0.186	-0.186	**0.788**	0.108
Ca	**0.845**	0.365	-0.146	0.208	0.127	0.022	0.031
Mg	**0.835**	0.371	-0.168	0.216	0.112	0.025	0.074
ESP	-0.205	-0.178	-0.036	-0.308	0.437	0.499	0.010
CEC	**0.897**	0.200	0.232	-0.047	0.130	0.007	0.123
Sand	**-0.858**	-0.038	-0.193	0.068	-0.314	-0.024	0.087
Silt	0.174	-0.054	0.325	0.235	**0.674**	0.183	-0.394
Clay	**0.905**	0.076	0.094	-0.183	0.099	-0.041	0.068
BD	**-0.927**	-0.063	-0.069	-0.012	-0.061	0.255	-0.010
AWC	**0.822**	0.182	0.184	-0.020	-0.313	0.161	0.198
**TProbe**	0.032	-0.012	0.437	**0.727**	0.169	0.119	0.068
Br_har	0.081	0.516	0.170	0.102	0.445	-0.096	0.492
**Pod_har**	0.318	0.320	**0.736**	0.242	0.117	-0.074	0.104

Bold typeface - Strong correlation coefficient TProbe: Surface soil moisture, Br_har: Number of branches at harvest, Pod_har:Number of pods at harvest, EC: electric conductivity, ESP: exchangeable sodium percentage, CEC: cation exchange capacity, BD: bulk density, AWC: available water content.

**Table 4.** Factor loadings of soil properties (before sowing during June 2019).

Parameters	PC1	PC2	PC3	PC4	PC5	PC6	PC7
Eigen value	7.567	2.743	2.288	2.146	2.102	1.689	1.653
% of Variance	30.269	10.971	9.154	8.586	8.408	6.754	6.612
Cumulative %	30.269	41.240	50.393	58.979	67.387	74.141	80.753
**KMO=0.555**							
pH	0.176	0.285	-0.052	**-0.614**	-0.167	0.372	0.422
EC	-0.022	0.116	0.084	**0.838**	-0.040	0.164	0.112
OC	0.405	0.529	0.100	**0.558**	0.348	-0.196	0.137
CaCO _3_	-0.001	**0.793**	-0.174	-0.110	0.144	-0.133	0.017
N	0.408	0.529	0.100	**0.557**	0.345	-0.198	0.137
**P _2_O _5_**	-0.351	-0.128	0.104	0.206	0.125	**-0.715**	0.349
**K _2_O**	**0.764**	-0.110	0.036	-0.062	0.281	0.174	0.358
**S**	-0.091	-0.271	0.089	0.120	-0.016	**0.719**	0.307
**Fe**	0.145	-0.056	**0.889**	0.030	-0.180	0.043	-0.024
**Zn**	-0.065	-0.065	**0.735**	0.138	0.163	-0.055	-0.367
**Mn**	**0.882**	0.243	0.182	-0.073	0.061	0.023	0.145
Cu	0.073	**0.764**	0.063	0.095	0.083	0.158	-0.244
B	-0.165	0.118	0.238	-0.109	-0.091	-0.013	**-0.726**
**Ca**	**0.900**	0.143	-0.153	0.196	0.074	-0.042	0.054
**Mg**	**0.919**	0.082	-0.207	0.153	0.014	-0.023	-0.014
ESP	-0.094	-0.036	-0.183	0.129	**-0.657**	0.495	-0.023
**CEC**	**0.937**	0.150	0.212	0.077	-0.012	0.030	-0.069
**Sand**	**-0.901**	0.051	-0.150	0.014	0.165	-0.087	-0.059
Silt	0.247	-0.209	0.306	-0.021	- **0.583**	0.096	0.377
**Clay**	**0.929**	0.042	0.047	0.000	0.031	0.065	-0.086
**BD**	**-0.743**	-0.017	-0.098	0.263	-0.120	0.056	-0.308
**AWC**	**0.625**	0.205	0.361	0.121	0.365	-0.128	0.078
TProbe	0.319	0.061	-0.066	0.241	**0.754**	0.084	0.217
Br_har	0.212	**0.595**	0.101	0.311	-0.343	-0.166	0.001
**Pod_har**	0.537	0.441	**0.605**	0.043	0.034	-0.126	0.165

**Bold**: Strong correlation coefficient TProbe: Surface soil moisture, Br_har: Number of branches at harvest, Pod_har: Number of pods at harvest, EC: electric conductivity, ESP: exchangeable sodium percentage, CEC: cation exchange capacity, BD: bulk density, AWC: avaialble water content.

In 2018 (site year 1), PC4 was identified as root proliferation stage, with high loadings of P _2_O _5_ (0.801) and surface soil moisture (0.727). Root interception of inorganic phosphorous increases with root proliferation. The diffusion of P _2_O _5_ from the soil through the roots increases with increased moisture content of dry soil ( Lambers *et al.*, 2006). Low rainfall ( Figure 2) events during critical growth stages of pigeon pea (September 2018 to December 2018), led to dry soil and reduced the dissolution of readily available (water-soluble) nutrients, which are required for plant growth. During the drought, when soil moisture slightly increased from an uneven and low precipitation pattern during November and December 2018, the plant enhanced its root proliferation in the presence of high available soil P _2_O _5_, in search of soil nutrients ( Basu *et al.*, 2016; Peret *et al.*, 2014). In 2019 (site year 2), PC4 was identified as humification with high loadings of EC (0.838), OC (0.558) and N (0.557) suggesting activation of humification ( Doni *et al.*, 2014). In site year 1, PC5 was identified as chemical changes in soil, and soil aeration supported by high loadings of soil pH (0.752) and silt (0.674). During site year 2, in PC5, soil ESP (-0.657) and silt (-0.583) showed a high negative factor loading, however available surface moisture (0.754) exhibited a high positive factor loading; this implies that when in water, dissociated calcium cations from CaCO _3_ in the presence of CO _2,_ have replaced sodium ions and adsorbed on the soil colloids ( van de Graaff & Patterson, 2001). In site year 1, PC6 had high negative factor loadings of CaCO _3_ (-0.781) and positive factor loadings of available boron (0.788), which are attributed to plant cell wall synthesis and root nodule formation for N _2_ fixation in leguminous crops. Growing plant cells responds well to boron for dividing and synthesizing cell walls when compared to growth-limited plant cells, in which the synthesis of primary cell walls is negligible ( Fleischer *et al.*, 1998). In leguminous plant roots, the development of symbiosis with soil *Rhizobium* bacteria depends on the concentration of available soil boron and calcium and that both nutrients are essential for nodule structure and fixing atmospheric nitrogen in the plant roots ( Redondo-Nieto *et al*., 2003). As CaCO _3_ is a source of calcium required by the plants during critical growth stages, the dissolution of CaCO _3_ in soil will be very rapid in the presence of carbon monoxide (CO), ( Gallagher & Breecker, 2020), therefore the negative factor loading of CaCO _3_ was observed in PC6 of site year 1. In PC6 of site year 2, high factor loadings of available P _2_O _5_ (-0.715) and sulphur (0.719) were found, whereas in PC7 of site year 1, available copper (0.682) and sulphur (-0.688) had high loadings associated with enhanced enzyme activity essential for nodule formation and nitrogen fixation in the leguminous plant roots ( Weisany *et al.*, 2013). PC7 of site year 2 showed a high negative factor loading of available boron (-0.726). Legume crops required more amount of boron compared to most field crops as boron plays a vital role in the development of reproductive organs, pollen germination, flower and fruit setting. Boron being a required trace element, there is a narrow gap between deficiency and toxicity in soil–plant systems ( Chatterjee & Bandyopandhyay, 2017). Therefore, one spray at the initiation of the reproductive phase is sufficient for optimum flowering and pod yield ( Subasinghe *et al*., 2003); without initial boron spray, plant uptake will deplete boron in the soil, hence the factor loading in PC7 being negative for boron.

All seven PCs of site year 1, when subjected to stepwise linear regression with crop yield, explained the variance with an R ^2 ^= 0.68 (p<0.01). The F test of ANOVA was significant at the 1% level. The coefficients of PC3 and PC4 were significant at the 1% level independently, whereas PC7 was significant at the 5% level. Therefore, the variables explaining PC3, PC4 and PC7 such as Fe, Zn, number of pods at harvest, available P _2_O _5_, soil moisture, and copper significantly influenced the yield ( Equation 3, Table 5). Available Zn and P _2_O _5_ help in seed formation, whereas available soil moisture supports crop growth, which was observed in terms of number of pods per plant and seed yield at the time of harvest. ( Subrahmaniyan *et al*., 2008).

**Table 5.** Estimations of soil properties and growth parameters of Kalmandari Tanda-1 MWS.

Independent variables	Dependent variable	Estimates					
		Principal components	Constant	Co-efficient	t	F	R ^2^
Surface soil and pigeon pea growth parameters	Y _seed,2018–19_	PC3	1300.913	0.636	4.898**	13.414**	0.679
		PC4		0.397	3.057**		
		PC7		0.341	2.628*		
	Y _seed,2019–20_	PC1	1421.000	0.698	5.349**	13.264**	0.677
		PC3		0.339	2.596**		
		PC6		-0.275	-2.106*		

**Y _seed_ = Crop yield**

Equation 3:

Y _seed_ = 1300.913 + 0.636 ^**^PC3 + 0.397 ^**^PC4 + 0.341 ^*^PC7, F=13.414 ^*^, R ^2^=0.679

All seven PCs of site year 2, when subjected to stepwise linear regression with crop yield, explained the variance significantly at the 1 % level ((R ^2^ = 0.677 p<0.01). The PC1 and PC3 coefficient was significant at the 1% level. PC6 coefficient was significant at 5 % level (p<0.05). Therefore, the variables constituting PC1, PC3 and PC6 such as K _2_O, S, Fe, Zn, Mn, Ca, Mg, CEC, clay, available water content and number of pods at harvest were significantly influencing the yield ( Equation 4, Table 5). This may be due to increased availability of nutrients such as K _2_O, Fe, Zn, Mn, and the presence of high clay content, which enhances the available water and water holding capacity of the soil; this facilitates nutrient uptake, leading to the increase in number of pods ( Rana & Badiyala, 2014; Zarei *et al*., 2011).

Equation 4:

Y _seed_ = 1421 + 0.698 ^**^PC _1_ + 0.339 ^**^PC3 - 0.275 ^*^PC6, F = 13.264 ^**^, R ^2 ^= 0.677

## Conclusion

In the present study, a systematic methodological approach was adopted, using geospatial and multivariate statistical techniques for spatial classification of soils, and to gauge the major variables influencing crop yield, which helps in deriving land- or soil-specific management interventions.

Our study revealed that the predominant yield-contributing variables in rainfed agriculture depended on a number of factors, including distribution of rainfall and prevailing land slope. During a drought year (site year 1), the availability of P _2_O _5_, Fe, Zn, Cu in the soil, and number of branches and pods determined the yield. In contrast, during a high-precipitation monsoon year (site year 2), availability of K _2_O, Mn, Ca, Mg, Fe, Zn, S, CEC, clay content, available water content and number of pods per plant were the main yield-contributing factors. Therefore, in a semi-arid region, an increase in soil moisture/available water content to an optimum level (25–30% AWC) will help dissolve most soil nutrients, and increases CEC and nutrient uptake, thus leading to a higher crop yield.

The principal component regression analysis performs a reasonable dimension reduction and operates well with highly correlated variables. Crop growth and yield depend on multiple factors but are limited by the least-available nutrient present in the environment relative to demands of this nutrient for growth. Therefore, all the loaded PCs with a minimum of 80 % cumulative variance should be considered for principal component regression analysis. The variables which were identified from the principal component regression analysis can efficiently explain the yield variability; further cross-verification of these variables with their availability in each soil management/soil-phase unit, may help to identify limiting factors, such as land surface parameters, soil depth, slope, and available soil moisture. Interventions involving these variables would need to be applied in a site-specific way to increase crop yield.

## Data Availability

Mendeley Data: Support for improved program integration under rainfed areas, https://data.mendeley.com/datasets/ng5v92xht3/4 ( Rajesh, 2021). This project contains the following underlying data: Table-1 2018–19-Before Sowing.xlsx (Physico-chemical properties of initial surface soil samples of Kalmandari Tanda-1 MWS during June 2018) Table-1-2019–20-After harvest.xlsx (Physico-chemical properties of surface soil samples (after harvest) of Kalmandari Tanda-1 MWS during 2018–19) Table-2-2018–19-Crop growth yield.xlsx (Pigeonpea plant growth and yield parameters of Kalmandari Tanda-1 MWS during kharif 2018–19) Table-2-2019–20-Crop growth yield.xlsx (Pigeonpea plant growth and yield parameters of Kalmandari Tanda-1 MWS during kharif 2019–20) Mendeley Data: Support for improved program integration under rainfed areas, https://data.mendeley.com/datasets/ng5v92xht3/4 ( Rajesh, 2021). This project contains the following extended data: Annexure-1.pdf (Pedon description form) Extended data-1.xlsx (pigeon pea growth variety and fertilizers used) Data are available under the terms of the Creative Commons Attribution 4.0 International license (CC-BY 4.0).
