# Supplementary material for: Variability in soil properties influencing pigeonpea ( Cajanus cajana L.) yield: a multivariate statistical analysis
Source: F1000Res. 2023 Aug 17;10:944. Originally published 2021 Sep 20. [Version 3] doi: 10.12688/f1000research.53095.3 (PMC10477742; doi:10.12688/f1000research.53095.3)
Supplement: Peer review history

# Peer review: Reviewer response for version 3

**DOI:** 10.5256/f1000research.151777.r197715

**Published:** 2023-09-04

**Authors:** Gabriel Oladele Awe

**Affiliations:** 1 Ekiti State University, Ado Ekiti, Nigeria

The authors have effected the corrections.

Is the work clearly and accurately presented and does it cite the current literature?

Yes

If applicable, is the statistical analysis and its interpretation appropriate?

Yes

Are all the source data underlying the results available to ensure full reproducibility?

Yes

Is the study design appropriate and is the work technically sound?

Yes

Are the conclusions drawn adequately supported by the results?

Yes

Are sufficient details of methods and analysis provided to allow replication by others?

Yes

Reviewer Expertise:

Soil and Water Conservation, Soil Physical processes, Irrigation, Soil tillage, Cropping systems

I confirm that I have read this submission and believe that I have an appropriate level of expertise to confirm that it is of an acceptable scientific standard.

# Peer review: Reviewer response for version 2

**DOI:** 10.5256/f1000research.144782.r173203

**Published:** 2023-06-01

**Authors:** Gabriel Oladele Awe

**Affiliations:** 1 Ekiti State University, Ado Ekiti, Nigeria

The authors have improved the manuscript, however some of the issues raised by this Reviewer were unattended to.

1. The Introduction still has some Methodology statements, not acceptable.
2. One Figure (Fig 6) still has an embedded table displaying same information.
3. Repetition of equation with same information in Table 5.

See attached PDF for further comments.

Is the work clearly and accurately presented and does it cite the current literature?

Yes

If applicable, is the statistical analysis and its interpretation appropriate?

Yes

Are all the source data underlying the results available to ensure full reproducibility?

Yes

Is the study design appropriate and is the work technically sound?

Yes

Are the conclusions drawn adequately supported by the results?

Yes

Are sufficient details of methods and analysis provided to allow replication by others?

Yes

Reviewer Expertise:

Soil and Water Conservation, Soil Physical processes, Irrigation, Soil tillage, Cropping systems

I confirm that I have read this submission and believe that I have an appropriate level of expertise to confirm that it is of an acceptable scientific standard, however I have significant reservations, as outlined above.

# Peer review: Reviewer response for version 1

**DOI:** 10.5256/f1000research.56445.r157225

**Published:** 2023-02-17

**Authors:** Gabriel Oladele Awe

**Affiliations:** 1 Ekiti State University, Ado Ekiti, Nigeria

The paper sought to evaluate the variability of soil properties using multivariate analysis with a view to detecting which properties influence the yield of pigeon pea.

 The study could contribute to knowledge in the area of site-specific agriculture. However, the Introduction requires thorough revision: it contains some statements that should appear in Methodology.

 A table (Table 3) appears repeating information already presented in the text. Also the Table inserted under Figure 6 is a repetition of same Figure, remove!

 For other comments, please refer to the text.

Is the work clearly and accurately presented and does it cite the current literature?

Yes

If applicable, is the statistical analysis and its interpretation appropriate?

Yes

Are all the source data underlying the results available to ensure full reproducibility?

Yes

Is the study design appropriate and is the work technically sound?

Yes

Are the conclusions drawn adequately supported by the results?

Yes

Are sufficient details of methods and analysis provided to allow replication by others?

Yes

Reviewer Expertise:

Soil and Water Conservation, Soil Physical processes, Irrigation, Soil tillage, Cropping systems

I confirm that I have read this submission and believe that I have an appropriate level of expertise to confirm that it is of an acceptable scientific standard, however I have significant reservations, as outlined above.

# Peer review: Reviewer response for version 1

**DOI:** 10.5256/f1000research.56445.r94826

**Published:** 2021-10-18

**Authors:** Sathish Ayyappa

**Affiliations:** 1 Department of Soil Science and Agricultural Chemistry, University of Agricultural Sciences, Bangalore, Bengaluru, India

The title of the article is precise and straight and does match with the research findings. Abstract and keywords are well framed with-in the 300 words. Methods adopted are very comprehensive and comprises a detailed soil survey at 1:8000 scale and classifying soils to series level and further dividing each series to soil phases/management units. Use of PCA and LPCR analysis to find out influence of soil properties on pigeonpea yield at soil phase level is also an indication of site specific management for various agronomic/soil operations. The technology adopted namely remote sensing data interpretation (for soil survey and classification), various lab standard analytical techniques/methods, GIS mapping, PCA, and LPCR are Apt. Therefore, the article can be approved for indexing with the following minor corrections; 

1. Page no. 4: last line of the first paragraph in the second column of the page “with standard HCl (Piper, 1966).” **w** letter is missing.
2. Page no. 12 & 13: Expansion of AWC, just below the table 4 and 5, should be changed to **Available **water content instead of Average water content.
3. Page No. 8: Under the sub heading ‘PCA & LPCR to assess the influence of soil properties on pigeonpea yield’ 2 ^nd^ column of the page no. 8, 7 ^th^ line, high loadings of clay content (0.897 and 0.937) these values should be changed to **(0.905 and 0.929) **as per the table 4 & 5 respectively.
4. Page No. 8: Under the sub heading ‘PCA & LPCR to assess the influence of soil properties on pigeonpea yield’ 2 ^nd^ column of the page no. 8, 9 ^th^ to 14th ^th^ line (on the other hand …..(Figure 2)). Please rewrite the sentence as given below;
  "High clay content supported high available water content with loadings of 0.822 and 0.625 during 2018-19 and 2019-20 respectively. Therefore, sand (-0.858 and -901) and bulk density (-0.927 and -0.743) are negatively correlated in PC1 of both the site years. Though the initial mean available water content is high (27.93 %) in soils during 2018-19 (Table 1), the scarcer rains in subsequent months during 2018-19 (Figure 2) has inferred that there was no sufficient soil moisture to dissolve the soil nutrients to make it readily available to pigeonpea plant when compared to the rains of 2019-20 (Figure 3)."
5. Page No. 13: 2 ^nd^ column, line 2, correct the spelling of ‘responds’.
6. Page 14: 2 ^nd^ column of the page, under equation 1, line 5, The PC1 and **PC3** coefficients **were** significant at 1 % (p<0.01) level. **PC6 was significant at 5 % (p<0.05)**. Note that PC3 significant is only at 1 % and not at 5 %.

Is the work clearly and accurately presented and does it cite the current literature?

Yes

If applicable, is the statistical analysis and its interpretation appropriate?

I cannot comment. A qualified statistician is required.

Are all the source data underlying the results available to ensure full reproducibility?

Yes

Is the study design appropriate and is the work technically sound?

Yes

Are the conclusions drawn adequately supported by the results?

Yes

Are sufficient details of methods and analysis provided to allow replication by others?

Yes

Reviewer Expertise:

NA

I confirm that I have read this submission and believe that I have an appropriate level of expertise to confirm that it is of an acceptable scientific standard.
